# The *ACTN3* R577X Polymorphism across Three Groups of Elite Male European Athletes

**DOI:** 10.1371/journal.pone.0043132

**Published:** 2012-08-16

**Authors:** Nir Eynon, Jonatan R. Ruiz, Pedro Femia, Vladimir P. Pushkarev, Pawel Cieszczyk, Agnieszka Maciejewska-Karlowska, Marek Sawczuk, Dmitry A. Dyatlov, Evgeny V. Lekontsev, Leonid M. Kulikov, Ruth Birk, David J. Bishop, Alejandro Lucia

**Affiliations:** 1 Institute of Sport, Exercise and Active Living (ISEAL), Victoria University, Victoria, Australia; 2 School of Sports and Exercise Sciences, Victoria University, Victoria, Australia; 3 Department of Physical Education and Sport, School of Sport Sciences, University of Granada, Granada, Spain; and Unit for Preventive Nutrition Department of Biosciences and Nutrition at NOVUM, Karolinska Institute, Huddinge, Sweden; 4 Department of Biostatistics, School of Medicine, University of Granada, Granada, Spain; 5 Ural State University of Physical Culture, Ural, Russia; 6 Department of Physical Culture and Health Promotion, University of Szczecin, Szczecin, Poland; 7 Department of Genetics, University of Szczecin, Szczecin, Poland; 8 Faculty of Health Sciences, Department of Nutrition, Ariel University Center, Ariel, Israel; 9 Universidad Europea de Madrid, Madrid, Spain; University of Zaragoza, Spain

## Abstract

The *ACTN3* R577X polymorphism (rs1815739) is a strong candidate to influence elite athletic performance. Yet, controversy exists in the literature owing to between-studies differences in the ethnic background and sample size of the cohorts, the latter being usually low, which makes comparisons difficult. In this *case:control* genetic study we determined the association between elite athletic status and the *ACTN3* R577X polymorphism within three cohorts of European Caucasian men, i.e. Spanish, Polish and Russian [633 cases (278 elite endurance and 355 power athletes), and 808 non-athletic controls]. The odds ratio (OR) of a power athlete harbouring the XX versus the RR genotype compared with sedentary controls was 0.54 [95% confidence interval (CI): 0.34–0.48; *P* = 0.006]. We also observed that the OR of an endurance athlete having the XX versus the RR genotype compared with power athletes was 1.88 (95%CI: 1.07–3.31; *P* = 0.028). In endurance athletes, the OR of a “world-class” competitor having the XX genotype versus the RR+RX genotype was 3.74 (95%CI: 1.08–12.94; *P* = 0.038) compared with those of a lower (“national”) competition level. No association (P>0.1) was noted between the *ACTN3* R577X polymorphism and competition level (world-class versus national-level) in power athletes. Our data provide comprehensive support for the influence of the *ACTN3* R577X polymorphism on elite athletic performance.

## Introduction

One of the most investigated genes with respect to elite athletic performance is the *ACTN3*. This gene encodes α-actinin-3, a sarcomeric protein that is almost exclusively expressed in fast, glycolytic, type ΙΙ fibres, where it plays an important role in the generation of ‘explosive’ powerful contractions [1], [2]. A nonsense polymorphism (rs1815739) was identified in the *ACTN3* gene, which results in replacement of an arginine (R) residue with a premature stop codon (X) at amino-acid 577 [3]. Approximately 18% of Caucasians are homozygous for the stop codon (i.e. they harbour the XX genotype) and are completely deficient in α-actinin-3 [1], [4].

The α-actinin-3 knock-out (KO) mouse model was subsequently developed to explore the biological role of the *ACTN3* R577X polymorphism [5], [6]. Compared with their wild- type counterparts, KO mice show lower and higher activity of the anaerobic and aerobic pathways respectively [6]. KO mice also exhibit higher time to fatigue, lower muscle mass and fibre diameter of fast-twitch (type IIB) muscle fibres, and lower muscular strength [5], [6]. These observations support the hypothesis that the *ACTN3* R577X polymorphism has functional significance in muscle function and metabolism, and might be influential to elite athletic performance. However, genetic association studies in cohorts of athletes have produced contradictory findings.

The *ACTN3* R allele, or the RR genotype, has been positively associated with elite, power-oriented athletic status (e.g. sprinters, jumpers or throwers) in some [4], [7], [8], [9], [10], [11], [12], but not all cohorts of Caucasian athletes [13], [14]. Similarly, the X allele or the XX genotype has been positively associated with elite endurance athletic status [4], [7], [12], although some studies have not observed a significant association with endurance phenotypes in non-athletic [15], [16] or athletic cohorts [17], [18], [19], [20], [21]. Some of the contradictory findings may be due to between-study differences in the competition level of the recruited athletes, which reflects the absence of a universally-accepted definition of what is considered ‘elite-level’. The inclusion of mixed-gender populations in previous studies may also have contributed to between-study differences.

The principal methodological limitation in the field of genetics and athletic performance is the need for large population samples (i.e. more than a few hundreds) to reach sufficient statistical power to allow making solid conclusions [22], [23]. Most of the aforementioned studies performed with elite athletes were limited by a relatively low sample size (n ≤200), which is understandable due to the low number of athletic champions available for analysis. Thus, multi-centre studies involving athletes from different nationalities are needed. In the present study, we were able to recruit over 600 elite male athletes from three different European countries in an attempt to overcome the sample size limitation.

Therefore, the main aim of the present study was to determine the association of the *ACTN3* R577X polymorphism with both elite endurance and power athletic status, in a large group of European Caucasians, consisting of Spanish, Polish and Russian athletic and non-athletic (controls) men. Additionally, we also examined the association of the *ACTN3* R577X polymorphism with the level (national-level vs. world-class) of athletic participation in both endurance and power athlete groups.

## Materials and Methods

The study was conducted according to the Declaration of Helsinki. Written informed consent was obtained from all participants, and the study was approved by the ethics committees of *Universidad Europea de Madrid*, Spain, the Pomeranian Medical University, Poland, and the Ural State University of Physical Culture, Russia.

### Participants

A total of 633 athletes (278 endurance athletes and 355 power athletes) and 808 controls, from Spain, Poland and Russia, volunteered to participate in this study. All participants were unrelated European males and all Caucasians (as self-reported) for ≥3 generations. We included athletes in the study sample only if they had participated in national/international championships and they had never tested positive in anti-doping controls. Inclusion criteria for all control participants were to be free of any diagnosed cardiorespiratory disease and not engaged in competitive sports or in formal, supervised exercise training (i.e. performing less than 3 structured weekly sessions of strenuous exercise such as running, swimming, bicycling or weight lifting).

#### Spanish population

The population comprised 273 elite athletes and 343 controls:

(i) 119 elite power athletes aged 20–33 years, including the best Spanish volleyball players (n = 66), track and field jumpers (n = 13) and sprinters (n = 40) in recent years. Thirteen track and field athletes were Olympians during the period 2000–2008.

(ii) 154 elite endurance athletes aged 20–39 years. This sample included 50 elite endurance runners (the top Spanish runners during the 1999–2009 period, i.e. mainly 5,000 m to marathon specialists, virtually all of them Olympians), 50 professional road cyclists who were all Tour de France finishers (including stage winners), and 54 rowers. A total of 39 rowers won at least one bronze, silver or gold medal in the lightweight category (skip or scull, including a total of 6 gold medals) in the World Championships held during 1997–2006.

(iii) 343 healthy, non-athletic controls aged 19–32 years. All were undergraduate students from the same university (*Universidad Europea de Madrid*, Spain).

#### Polish population

The population comprised 217 elite Polish athletes and 354 controls:

(i) 105 elite power athletes. This group included weightlifters (n = 49, including 2 Olympic champions, 3 World champions (power-lifting) and 10 medallists in World or European championships), sprinters (≤200 m, n = 46, including an Olympic champion and 9 medallists in Olympic games or World/European championships), track and field jumpers (n = 6, including an Olympic champion, and 3 medallists in Olympic Games or World/European championships), and volleyball players (n = 4, all medallists in Olympic Games or World/European championships).

(ii) 112 elite endurance athletes. This group included rowers (n = 53, including 14 Olympic/Word champions and 22 medallists in Olympic Games or World/European championships), endurance road cyclists (n = 14, including 7 medallists in Olympic Games or World/European championships), 5,000 m runners (n = 12, including 1 Olympic medallist), marathon runners (n = 12), 800–1,500 m swimmers (n = 11, including 2 medallists in Olympic Games or World/European championships)), 15–50 km cross-country skiers (n = 6, including 2 Olympic champions), and triathletes (n = 4, all are silver or bronze medallist in European championships).

(iii) 354 healthy sedentary controls aged 19–32 years (all students of the University of Szczecin).

#### Russian population

The population sample comprised 143 Russian athletes, and 111 controls:

(i) 125 elite power athletes. This group included: ice hockey players of the *Kontinental Hockey League* (KHL), which is the highest ranked hockey league in Europe (n = 59), skaters competing in events ≤1000 m (n = 17, including 3 World champions and 3 European champions), boxers (n = 18, including 8 World champions and 3 European champions), wrestlers (n = 10, including 3 European champions), swimmers competing in events ≤200 m (n = 8), weightlifters (n = 6, including the World Powerlifting Congress record man), figure skaters (n = 3), shot putters (n = 2), one strongman (vice-world champion and three times Russia’s Strongest Man), and one taekwondo specialist.

(ii) 18 elite endurance athletes. This group included rowers (n = 6), skaters competing in events ≥5000 m (n = 4), walkers (n = 4, including one winner of the European Cup), one swimmer competing in events >200 m (bronze medallist in European championships, Olympian in 2008), one marathon runner (European champion), one duathlete, and one water polo player.

According to their individual best performances, we further divided the athletes within each group into two subgroups: ‘world-class’, i.e. those who had represented their country in European or World championships, or in the Olympic Games; and ‘national-level’, i.e. those who had competed at a national but not international level (Table 1).

**Table 1:** Genotype and allele frequencies of the *ACTN3* R577X polymorphism (rs1815739) in male endurance and power athletes, and in male sedentary controls, from Spain, Poland and Russia.

	Spain (n = 616)				Poland (n = 571)				Russia (n = 254)			
	Control	Endurance	Power	*P* *	Control	Endurance	Power	*P* *	Control	Endurance	Power	*P* *
*all*	343	154	119	0.119	354	112	105	0.465	111	18	125	0.019
RR	106 (30.9)	41 (26.6)	37 (31.1)		140 (39.5)	46 (41.1)	46 (43.8)		39 (35.1)	8 (44.4)	43 (34.4)	
RX	175 (51)	73 (47.4)	66 (55.5)		176 (49.7)	56 (50.0)	54 (51.4)		46 (41.4)	10 (55.6)	68 (54.4)	
XX	62 (18.1)	40 (26.0)	16 (13.4)		38 (10.7)	10 (8.9)	5 (4.8)		26 (23.4)	0 (0.0)	14 (11.2)	
MAF	0.436	0.497	0.412		0.356	0.339	0.305		0.441	0.278	0.384	
HWE-P value	0.564	0.938	0.004		0.134	0.290	0.044		0.124	0.242	0.125	
*World-class*		139	119	0.022		68	66	0.203		11	89	0.732
RR		38 (27.3)	37 (31.1)			32 (47.1)	25 (37.9)			5 (45.5)	34 (38.2)	
RX		63 (45.3)	66 (55.5)			27 (39.7)	36 (54.5)			6 (54.5)	45 (50.6)	
XX		38 (27.3)	16 (13.4)			9 (13.2)	5 (7.6)			0 (0.0)	10 (11.2)	
MAF		0.500	0.412			0.331	0.349			0.273	0.365	
*National-level*		15	–	–		44	39	0.069		7	36	0.569
RR		3 (20.0)	–			14 (31.8)	21 (53.8)			3 (42.9)	9 (25.0)	
RX		10 (66.7)	–			29 (65.9)	18 (46.2)			4 (57.1)	23 (63.9)	
XX		2 (13.3)	–			1 (2.3)	0 (0.0)			0 (0.0%)	4 (11.1)	
MAF		0.467				0.352	0.231			0.286	0.431	

* Fisher’s exact test.

Values are frequency (percent).

HWE: Hardy–Weinberg equilibrium; MAF: minor allele frequency.

### Genotyping

We followed recent recommendations for genotype-phenotype association studies provided by Chanock et al. [24], Attia et al. [25], and the latest ‘Strengthening the Reporting of Genetic Association studies’ (STREGA) group report [26].

#### Spanish population

Genomic DNA was isolated from buccal epithelium or peripheral blood during the years 2004–2008 and genotyping was performed in the Genetics Laboratory of *Universidad Europea de Madrid*, Spain. We followed the *ACTN3* R577X genotyping methods that were applied in previous research [27]. The polymerase chain reaction (PCR) was performed in order to amplify the sequence containing the mutation. A fragment of 291 bp was amplified with the following primers: ACTN3-F 5′- CTGTTGCCTGTGGTAAGTGGG labelled a 5′ with VIC and ACTN3-R 5′- TGGTCACAGTATGCAGGAGGG. The PCR conditions were as follows: initial denaturing at 95°C 5 min; 35 cycles at 95°C 30 s, 60°C 30 s, 72°C 30 s and a final extension at 72°C 10 min. *ACTN3* genotypes were established by enzymatic digestion of amplicons with *DdeI*. The 25 µl reaction mix consisted of 5 µl aliquot amplified PCR product, 1X reaction NEB 3 Buffer (New England Biolabs, Beverly, MA, USA) and 1U of *DdeI* restriction enzyme (New England Biolabs, Beverly, MA, USA). The reaction mix was incubated 1 h at 37°C. The R577X change creates a restriction site resulting in fragments of 108, 97 and 86 bp. Digestion of the R577 allele results in fragments of 205 and 86 bp, and digestion of the 577X allele results in fragments of 108, 97 and 86 bp. The digestion products detected by capillary electrophoresis (ABI Prism 310 genetic analyzer; Applied Biosystems, Foster City, CA) were those labelled with VIC, i.e. 108 bp for 577X, and 205 bp for R577.

Following recent recommendations [24], we replicated the genotype results of the Spanish cohort (in 40% of samples) in another laboratory (*Progenika Biopharma, Parque Tecnológico de Zamudio*, Vizcaya, Spain) using a different method, i.e. a newly developed low-density DNA microarray based on allele-specific probes. The design, fabrication, validation and analysis of the arrays were performed following the procedure detailed elsewhere [28] with minor modifications. The PCR products were fluorescently labelled and hybridized to the DNA microarray in an automated platform (Tecan HS4800, Mannedorf, Switzerland). Finally, the microarrays were scanned (Innopsys S.A., Carbonne, France) and a software was used which converts the intensity of the spots into the genotype of the polymorphism.

**Table 2:** The odds ratio of *ACTN3* R577X (rs1815739) genotypes for endurance and power athletes.

	Controls vs. Endurance vs.	Controls vs. Power	Power vs. Endurance
RR (ref.)	1.0	1.0	1.0
RX	1.02 (0.75; 1.39)	1.07 (0.81; 1.41)	1.00 (0.69; 1.46)
P	0.897	0.655	0.982
XX	1.18 (0.78; 1.79)	0.54 (0.34; 0.84)	1.88 (1.07; 3.31)
P	0.424	0.006	0.028
RX+XX vs. (RR ref.)	1.06 (0.79; 1.42)	0.93 (0.71; 1.22)	1.14 (0.79; 1.63)
P	0.704	0.617	0.488
XX vs. RR+RX (ref.)	1.169 (0.81; 1.69)	0.52 (0.34; 0.78)	1.89 (1.12;3.14)
P	0.406	0.002	0.016

Data are odds ratio and 95% confidence intervals.

Analysis adjusted by country. Abbreviation: ref., reference group.

**Table 3:** The odds ratio of *ACTN3* R577X (rs1815739) genotypes for endurance and power athletes, according to their competition level (world-class vs. national-level).

	Endurance	Power
RR (ref.)	1.0	1.0
RX	0.41 (0.21; 0.82)	1.05 (0.60; 1.85)
P	0.011	0.864
XX	2.11 (0.28; 8.00)	1.99 (0.61; 6.55)
P	0.272	0.257
RX+XX vs. (RR ref.)	0.52 (0.26; 1.02)	1.13 (0.65; 1.96)
P	0.058	0.667
XX vs. RR+RX (ref.)	3.74 (1.08; 12.94)	1.94 (0.62; 6.07)
P	0.038	0.258

Data are odds ratio and 95% confidence intervals.

Analysis adjusted by country. Abbreviation: ref., reference group.

#### Polish population

Genomic DNA was isolated from buccal epithelium using GenElute Mammalian Genomic DNA Miniprep Kit (Sigma, Germany), during the years 2008–2010, according to the producer protocol. Genotyping was performed using polymerase chain reaction (PCR) as described elsewhere [29]. The amplified fragment subsequently underwent digestion by *DdeI*. The digested products were separated by 3% agarose gel electrophoresis, stained with ethidium bromide, and visualized in UV light.

**Figure 1:**
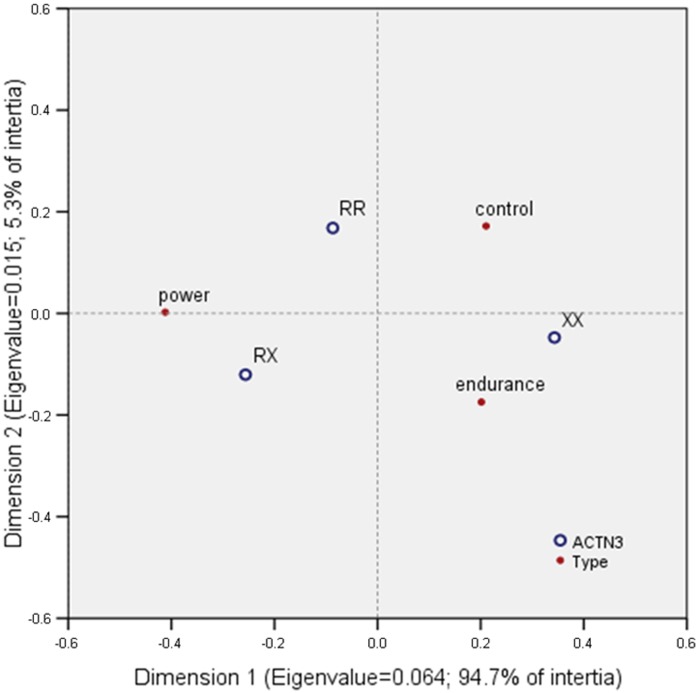
Correspondence diagram of athlete type with ACTN3 R577X (rs1815739) polymorphism.

#### Russian population

Genomic DNA was isolated from buccal epithelium or peripheral blood, during the years 2009–2011, using the Diatom™ DNA Prep kit (Cat. # D 1025, IsoGene Lab Ltd, Russia). The kit is based on selective DNA absorption on a surface of glass powder in the presence of high concentration of guanidine isothiocyanate as chaotropic agent.

Genotyping of the *ACTN3* R577X polymorphism was performed by using a TaqMan® SNP Genotyping Assay (Applied Biosystems, Foster city, CA, USA) with a StepOne™ Real-Time PCR System (Applied Biosystems, Foster city, CA, USA). Assay ID was C____590093_1_. The results were analyzed by using TaqMan® Genotyper Software (Applied Biosystems, Foster city, CA, USA). For replication purpose, 75% of the samples in the Russian cohort were analysed with a different method, i.e. PCR-restriction length polymorphism (RFLP), according to a previously described method [29]. The oligonucleotide primers for this method were synthesized by Evrogen Ru JSC (Russia). K562 DNA High Molecular Weight from Promega Corp. (Cat # DD2011, Madison, WI, USA) served as positive control samples at carrying out both research methods. Genetic profile of K562 DNA was XX in the *ACTN3* R577X sequence variation.

### Statistical Analysis

We used the χ^2^ test to test for the presence of Hardy–Weinberg equilibrium (HWE). We compared genotypic and allele frequencies in all groups using the Fisher’s exact test, as an omnibus test, separately by country (Spain, Poland and Russia). We conducted logistic regression analysis to analyse the association between: (i) *ACTN3* R577X polymorphism and athletic status (i.e. power athlete, endurance athlete, or non-athletic control), and (ii) *ACTN3* R577X polymorphism and competition level (national level or world-class) within each group of power or endurance athletes. Analyses were adjusted for country of origin. The association of the polymorphisms with athletic status was evaluated by conducting the following contrasts: RR (reference group) vs. RX, and RR vs. XX (co-dominant effect); RR vs. RX and XX combined (dominant effect); RR and RX combined (reference group) vs. XX (recessive effect). In addition, we conducted correspondence analysis with χ^2^ distance and symmetric normalization methods in order to graphically demonstrate the association between *ACTN3* R577X and athletic status.

All statistical analyses were conducted using the IBM-SPSS (v. 20.0 for WINDOWS, Chicago). A nominal *P-*value ≤0.05 was considered significant.

## Results

Replication analysis of the Spanish and the Russian samples using a different genotyping method was 100% successful. Table 1 show the genotype and allele frequencies of the *ACTN3* R577X polymorphism in all participants. Genotype distributions of all control and athletic groups were in agreement with the Hardy Weinberg Equilibrium (all *P*>0.1), except for the Spanish and Polish power athletes (*P* = 0.004 and *P* = 0.044 respectively).

Table 2 displays the association of the *ACTN3* R577X polymorphism with athletic status. The odds ratio (OR) of a power athlete harbouring the XX vs. the RR genotype (co-dominant effect) compared with sedentary controls was 0.54 [95% confidence interval (CI): 0.34–0.48; *P* = 0.006]. The results remain significant when the analysis was further adjusted by type of sport [OR: 0.33, 95% CI: 0.12–0.93; *P* = 0.036]. The OR of having the XX genotype vs. having the recessive trait (RR+RX combined) was 0.52 (95%CI: 0.34–0.78; *P* = 0.002), whereas the association was attenuated once the analysis was adjusted by type of sport (OR: 0.68, 95%CI: 0.29–1.59; *P* = 0.379). Compared with controls, the OR for an endurance athlete to harbour the XX vs. the RR genotype (co-dominant effect) was 1.18 (95%CI: 0.78–1.79; *P* = 0.424), whereas the OR of having the XX genotype vs. having the recessive trait (RR+RX combined) was 1.17 (95%CI: 0.81–1.69; *P* = 0.406). However, when the analyses were adjusted by type of sport the association became significant (OR: 5.02, 95%CI: 1.89–13.33, *P* = 0.001; and OR: 1.87, 95%CI: 1.16–3.01, *P* = 0.010). Compared with power athletes, the OR for an endurance athlete to harbour the XX vs. the RR genotype (co-dominant effect) was 1.88 (95%CI: 1.07–3.31; *P* = 0.028), whereas the OR of having the XX genotype vs. having the recessive trait (RR+RX combined) was 1.89 (95%CI: 1.12–3.14; *P* = 0.016). Similar results were observed after further adjusting by type of sport (OR: 3.33, 95%CI: 1.42–7.8, *P* = 0.005; and OR: 2.41, 95%CI: 1.12–5.15, *P* = 0.023, respectively). The findings did not materially change when the analyses were additionally adjusted by the athlete’s level of participation (world-class vs. national-level) (data not shown).

Table 3 shows the association between the *ACTN3* R577X polymorphism and competition level (national-level vs. world-class), within each group of endurance and power athletes. In endurance athletes, the OR of a world-class athlete having the XX genotype vs. having the recessive trait was 3.74 (95%CI: 1.08–12.94; *P* = 0.038) compared with a national-level athlete. However, the association was slightly attenuated once the analysis was further adjusted by type of sport (OR: 3.28, 95%CI: 0.79–13.62; *P* = 0.102). In power athletes, we observed no association (P>0.1) between the *ACTN3* R577X polymorphism and competition level (national-level vs. world-class).

Figure 1 graphically shows the correspondence analysis of athletic status with *ACTN3* R577X polymorphism. The first dimension (i.e. dimension 1, horizontal line) observed accounted for 94.7% of the total inertia, which indicates that this dimension almost accounted for all the inertia of the observed data. This dimension was mainly generated by type =  power and by *ACTN3*genotype =  XX, which accounted for 66.7% and 61.7% respectively, of the total inertia of this dimension. The power athletes group and the XX genotype were displayed in the extremes of the figures, whereas the endurance athletes group was close to the XX genotype. The percentages of explained variance of each dimension by the categories are presented in a supplementary file (Table S1).

## Discussion

We studied the association between the *ACTN3* R577X polymorphism and elite athletic status in a large group of elite male athletes, comprising three cohorts of European Caucasian male athletes. The main findings of the present study were: (i) elite power athletes were less likely (∼50%) to harbour the XX genotype compared with sedentary controls; and ii) the *ACTN3* XX genotype was more prevalent in endurance athletes, who were 1.88 times more likely to harbour the XX genotype compared with power athletes, which was further strengthened after adjusting by type of sport (OR: 3.33). The potential favourable effect of the XX genotype on elite endurance athletic status was additionally supported by the finding that the chance of a world-class athlete to have the XX genotype vs. the other genotypes was ∼3.7 higher compared with a less successful (national-level) athletes. However, it must be emphasized that no Russian endurance athlete had the XX genotype. Reasons for this striking finding are unclear, although the small sample size of this specific athlete’s group within the Russian cohort might be a potential confounder. An additional reason might lie in the lack of athletes with a ‘pure’ endurance phenotype (e.g. marathoners, cross-country skiers or triathletes) in the Russian cohort.

We believe that the results of our study are overall valid, as all of the following criteria were met [25]: cases (athletes) clearly presented the main study phenotype (i.e. being an elite athlete); we studied some of the best elite endurance athletes world-wide, participants within each cohort were ethnically-matched; genetic assessment was accurate and unbiased; and genotype distributions were in HWE in the control group of the three cohorts. With regards to the fact that HWE was not met in the Spanish and Polish power athletes’ groups, in must be emphasized that, for a genetic association study with a case-control design as the present one, attainment of HWE should only be a requirement (and strictly speaking should only be tested) in the control group, because they are supposedly representative of the general population.

A novel finding of the present study is that the XX genotype is even more favourable for world-class endurance athlete status compared with national-level endurance athlete status. To date, only one study, with Israeli athletes [7], showed an association between the *ACTN3* R577X and elite athletic status with respect to the competition level of the athlete. However, the relatively small number of elite endurance athletes recruited for the aforementioned study (n = 20) made it difficult to reach a solid conclusion. Here, the sub-groups of world-class and national-level endurance athletes had a larger sample size (n = 218 and n = 66 respectively), providing support for an association between the *ACTN3* XX genotype and world-class endurance athlete status. This observation indicates that while this genotype is important in the development of endurance ability, it might be even more important in the development of world-class endurance ability.

Mechanistic observations using the α-actinin-3 deficient (KO) mouse model (i.e. with the XX genotype) provide support for our results. KO mice show significantly higher activity of mitochondrial enzymes, and longer treadmill running, compared with wild-type mice [1], [6]. Isolated KO mice muscles have longer twitch half-relaxation times and enhanced recovery from fatigue compared with muscles from wild-type animals, providing a possible explanation for poorer sprint and improved endurance performance in XX humans [1], [5], [6]. Further support for the association between the *ACTN3* R577X polymorphism and athletic status relies on the finding that humans with the XX genotype tend to have a higher percentage of slow-twitch (type I) muscle fibres, which would benefit endurance performance, whereas RR homozygotes have a higher percentage of fast-twitch (IIX) glycolytic muscle fibres, which might favour power-oriented performance [30]. However, others found that α-actinins do not play a significant role in determining human skeletal muscle fibre composition [31]. In KO mice, the cross-sectional area of fast-twitch (IIB) fibres is 34% smaller compared with wild-type mice [5]. Interestingly, a smaller diameter of human fast-twitch (IIX) fibres has been previously correlated with time-trial performance in endurance athletes (cyclists) [32]. A possible explanation is that a small diameter of IIX fibres may allow for a greater capillary density (capillaries/mm^2^) in these cells, as well as for an enhanced removal of lactate and H^+^, which would benefit endurance performance.

A favourable genetic endowment seems necessary to become an elite athlete, yet the putative influence of specific candidate genes on the possibility of becoming an athletic champion, whether individually or in combination with other variants or environmental factors, requires further investigation. The *ACTN3* R577X polymorphism is probably the most promising candidate gene among all of the studied athletic status-related genes, and the only muscle-structural gene showing a genotype:performance association. Taken together, and despite some controversy, most notably in East-African elite distance runners [21], present and previous findings support an overall favourable association between the *ACTN3* XX genotype and human endurance performance, at least in those endurance events where the ability to produce explosive peak power is not a main success determinant.

In conclusion, our findings provide support for an association between *ACTN3* R577X polymorphism and elite athletic status in a large group of elite male athletes. The findings indicated that the *ACTN3* XX genotype is more prevalent in endurance athletes, who are more likely to harbour this genotype compared with power athletes. Future studies are encouraged to recruit large enough samples of elite athletes from different ethnic and geographic backgrounds. Therefore, large collaborations and data sharing between researchers, as presented here, are strongly recommended. Mechanistic approaches are also encouraged to explain the observed genotype:phenotype associations.

## Supporting Information

Table S1 **Percent of explain variance of each dimension to the categories.**
(DOC)

## References

[1] MacArthur DG, North KN (2007) ACTN3: A genetic influence on muscle function and athletic performance. Exerc Sport Sci Rev 35: 30–34. 17211191 10.1097/JES.0b013e31802d8874

[2] MacArthur DG, North KN (2004) A gene for speed? The evolution and function of alpha-actinin-3. BioEssays 26: 786–795. 15221860 10.1002/bies.20061

[3] North KN, Yang N, Wattanasirichaigoon D, Mills M, Easteal S, et al (1999) A common nonsense mutation results in alpha-actinin-3 deficiency in the general population. Nat Genet 21: 353–354. 10192379 10.1038/7675

[4] Yang N, MacArthur DG, Gulbin JP, Hahn AG, Beggs AH, et al (2003) ACTN3 genotype is associated with human elite athletic performance. Am J Hum Genet 73: 627–631. 12879365 10.1086/377590 PMC1180686

[5] MacArthur DG, Seto JT, Chan S, Quinlan KG, Raftery JM, et al (2008) An Actn3 knockout mouse provides mechanistic insights into the association between alpha-actinin-3 deficiency and human athletic performance. Hum Mol Genet 17: 1076–1086. 18178581 10.1093/hmg/ddm380

[6] MacArthur DG, Seto JT, Raftery JM, Quinlan KG, Huttley GA, et al (2007) Loss of ACTN3 gene function alters mouse muscle metabolism and shows evidence of positive selection in humans. Nat Genet 39: 1261–1265. 17828264 10.1038/ng2122

[7] Eynon N, Duarte JA, Oliveira J, Sagiv M, Yamin C, et al (2009) ACTN3 R577X polymorphism and Israeli top-level athletes. Int J Sports Med 30: 695–698. 19544227 10.1055/s-0029-1220731

[8] Papadimitriou ID, Papadopoulos C, Kouvatsi A, Triantaphyllidis C (2008) The ACTN3 gene in elite Greek track and field athletes. Int J Sports Med 29: 352–355. 17879893 10.1055/s-2007-965339

[9] Gineviciene V, Pranculis A, Jakaitiene A, Milasius K, Kucinskas V (2011) Genetic Variation of the Human ACE and ACTN3 Genes and their Association with Functional Muscle Properties in Lithuanian Elite Athletes. Medicina 47: 284–290. 21956137

[10] Ahmetov II, Druzhevskaya AM, Lyubaeva EV, Popov DV, Vinogradova OL, et al (2011) The dependence of preferred competitive racing distance on muscle fibre type composition and ACTN3 genotype in speed skaters. Exp physiol 96: 1302–1310. 21930675 10.1113/expphysiol.2011.060293

[11] Roth SM, Walsh S, Liu D, Metter EJ, Ferrucci L, et al (2008) The ACTN3 R577X nonsense allele is under-represented in elite-level strength athletes. Eur J Hum Genet 16: 391–394. 18043716 10.1038/sj.ejhg.5201964 PMC2668151

[12] Niemi AK, Majamaa K (2005) Mitochondrial DNA and ACTN3 genotypes in Finnish elite endurance and sprint athletes. Eur J Hum Genet 13: 965–969. 15886711 10.1038/sj.ejhg.5201438

[13] Lucia A, Olivan J, Gomez-Gallego F, Santiago C, Montil M, et al (2007) Citius and longius (faster and longer) with no alpha-actinin-3 in skeletal muscles? Br J Sports Med 41: 616–617. 17289854 10.1136/bjsm.2006.034199 PMC2465381

[14] Druzhevskaya AM, Ahmetov II, Astratenkova IV, Rogozkin VA (2008) Association of the ACTN3 R577X polymorphism with power athlete status in Russians. Eur J Appl Physiol 103: 631–634. 18470530 10.1007/s00421-008-0763-1

[15] Vincent B, Windelinckx A, Van Proeyen K, Masschelein E, Nielens H, et al. (2011) Alpha-actinin-3 deficiency does not significantly alter oxidative enzyme activity in fast human muscle fibres. Acta physiol. doi: 10.1111/j.1748–1716.2011.02366.x. 10.1111/j.1748-1716.2011.02366.x 21933355

[16] Ruiz JR, Fernandez Del Valle M, Verde Z, Diez-Vega I, Santiago C, et al (2010) ACTN3 R577X polymorphism does not influence explosive leg muscle power in elite volleyball players. Scan J Med Sci Sports 21: e34–41. 10.1111/j.1600-0838.2010.01134.x 20561285

[17] Ahmetov II, Druzhevskaya AM, Astratenkova IV, Popov DV, Vinogradova OL, et al (2010) The ACTN3 R577X polymorphism in Russian endurance athletes. Br J Sports Med 44: 649–652. 18718976 10.1136/bjsm.2008.051540

[18] Doring FE, Onur S, Geisen U, Boulay MR, Perusse L, et al (2010) ACTN3 R577X and other polymorphisms are not associated with elite endurance athlete status in the Genathlete study. J Sports Sci 28: 1355–1359. 20845221 10.1080/02640414.2010.507675

[19] Muniesa CA, Gonzalez-Freire M, Santiago C, Lao JI, Buxens A, et al (2010) World-class performance in lightweight rowing: is it genetically influenced? A comparison with cyclists, runners and non-athletes. Br J Sports Med 44: 898–901. 18801770 10.1136/bjsm.2008.051680

[20] Saunders CJ, September AV, Xenophontos SL, Cariolou MA, Anastassiades LC, et al (2007) No association of the ACTN3 gene R577X polymorphism with endurance performance in Ironman Triathlons. Ann Hum Genet 71: 777–781. 17627799 10.1111/j.1469-1809.2006.00385.x

[21] Yang N, MacArthur DG, Wolde B, Onywera VO, Boit MK, et al (2007) The ACTN3 R577X polymorphism in East and West African athletes. Med Sci Sports Exerc 39: 1985–1988. 17986906 10.1249/mss.0b013e31814844c9

[22] Bouchard C (2011) Overcoming barriers to progress in exercise genomics. Exer Sport Sci Rev 39: 212–217. 10.1097/JES.0b013e31822643f6 PMC3183378 21697717

[23] Eynon N, Ruiz JR, Oliveira J, Duarte JA, Birk R, et al (2011) Genes and elite athletes: a roadmap for future research. J Physiol 589: 3063–3070. 21540342 10.1113/jphysiol.2011.207035 PMC3145924

[24] Chanock SJ, Manolio T, Boehnke M, Boerwinkle E, Hunter DJ, et al (2007) Replicating genotype-phenotype associations. Nature 447: 655–660. 17554299 10.1038/447655a

[25] Attia J, Ioannidis JP, Thakkinstian A, McEvoy M, Scott RJ, et al (2009) How to use an article about genetic association: B: Are the results of the study valid? JAMA 301: 191–197. 19141767 10.1001/jama.2008.946

[26] Little J, Higgins JP, Ioannidis JP, Moher D, Gagnon F, et al (2009) Strengthening the reporting of genetic association studies (STREGA): an extension of the STROBE Statement. Hum Genet 125: 131–151. 19184668 10.1007/s00439-008-0592-7

[27] Gomez-Gallego F, Santiago C, Gonzalez-Freire M, Muniesa CA, Fernandez Del Valle M, et al (2009) Endurance performance: genes or gene combinations? Int J Sports Med 30: 66–72. 18651373 10.1055/s-2008-1038677

[28] Tejedor D, Castillo S, Mozas P, Jimenez E, Lopez M, et al (2005) Reliable low-density DNA array based on allele-specific probes for detection of 118 mutations causing familial hypercholesterolemia. Clin Chem 51: 1137–1144. 15890894 10.1373/clinchem.2004.045203

[29] Mills M, Yang N, Weinberger R, Vander Woude DL, Beggs AH, et al (2001) Differential expression of the actin-binding proteins, alpha-actinin-2 and -3, in different species: implications for the evolution of functional redundancy. Hum Mol Genet 10: 1335–1346. 11440986 10.1093/hmg/10.13.1335

[30] Vincent B, De Bock K, Ramaekers M, Van den Eede E, Van Leemputte M, et al (2007) ACTN3 (R577X) genotype is associated with fiber type distribution. Physiol Genomics 32: 58–63. 17848603 10.1152/physiolgenomics.00173.2007

[31] Norman B, Esbjornsson M, Rundqvist H, Osterlund T, von Walden F, et al (2009) Strength, power, fiber types, and mRNA expression in trained men and women with different ACTN3 R577X genotypes. J Appl Physiol 106: 959–965. 19150855 10.1152/japplphysiol.91435.2008

[32] Bishop D, Jenkins DG, McEniery M, Carey MF (2000) Relationship between plasma lactate parameters and muscle characteristics in female cyclists. Med Sci Sports Exerc 32: 1088–1093. 10862534 10.1097/00005768-200006000-00008

