# Correction: The *ACTN3* R577X Polymorphism across Three Groups of Elite Male European Athletes

**DOI:** 10.1371/journal.pone.0093165

**Published:** 2014-03-17

**Authors:** The PLOS ONE Staff

It has come to our attention that the Academic Editor who handled this manuscript, Dr Nuria Garatachea, has collaborated in recent publications with the authors of the article.

In line with the *PLOS ONE* competing interest policy (http://www.plosone.org/static/competing.action), we consider this as a potential conflict of interest.

In the light of this potential competing interest, the *PLOS ONE* editors have asked an independent member of the editorial board to carefully evaluate the peer review process of this article. This adviser considered that it is necessary to correct the p value reported in the article and to provide additional clarification regarding the methodology and the limitations of the study.

The authors would therefore like to make the following corrections and additions to the article:

1. The P-value for the Spanish Power athletes in the published manuscript is 0.004, whereas it should be 0.150, using the Yates' correction. The change in P-value has no effect on the study results.

2. Figure 1 was generated using the correspondence analysis (CA), the figure is the perceptual map of CA. This approach is conceptually similar to the principal component analysis but applies to categorical rather than quantitative data. The rationale behind CA is the decomposition of the chi-squared statistic associated with a contingency table into orthogonal factors and its goal is to make bi-dimensional plots for contingency tables.

3. In paragraph 3 of the Introduction section we mentioned that "The ACTN3 R allele, or the RR genotype, has been positively associated with elite, power-oriented athletic status (e.g. sprinters, jumpers or throwers) in some [4,7,8,9,10,11,12], but not all cohorts of Caucasian athletes [13,14]'. We wish to add that some of these papers also mentioned that the XX genotype is underrepresented (or 'less frequent') in power athletes (references 4, 7, 8 in the article), and it therefore can also be hypothesized that the XX genotype would be underrepresented (or 'less frequent') in sprint/power athletes compared to the endurance athletes and the controls group.

4. As stated in the article, all athletes and controls are Caucasian for three generations and have been sampled from Spain, Russia and Poland, however the lack of ancestry informative markers to validate the matching on ethnicity constitutes a limitation of this study.
