# Supplementary material for: The ACTN3 R577X Polymorphism across Three Groups of Elite Male European Athletes
Source: PLoS One. 2012 Aug 16;7(8):e43132. doi: 10.1371/journal.pone.0043132 (PMC3420864; doi:10.1371/journal.pone.0043132)
Supplement: Table S1 — Percent of explain variance of each dimension to the categories. (DOC) [file pone.0043132.s001.doc]

Supplementary Table

**Table S1**. Percent of explain variance of each dimension to the categories.

|  | Dimension 1 | Dimension 2 | Total |
| --- | --- | --- | --- |
| **ATCN3** |  |  |  |
| RR | 53.1% | 46.9% | 100.0% |
| RX | 95.0% | 5.0% | 100.0% |
| XX | 99.6% | 0.4% | 100.0% |
| **Type** |  |  |  |
| Control | 86.4% | 13.6% | 100.0% |
| Endurance | 85.0% | 15.0% | 100.0% |
| Power | 100.0% | 0.0% | 100.0% |
